# Determination of the Contributing Factors and HbA1c Cutoff Leading to Glucose Tolerance Abnormalities Following Gestational Diabetes

**DOI:** 10.7759/cureus.56218

**Published:** 2024-03-15

**Authors:** Ach Taieb, Marwa Majdoub, Nesrine Souissi, Souhir Chelly, Asma Ben Abdelkrim

**Affiliations:** 1 Endocrinology, Hospital University of Farhat Hached Sousse Tunisia, Sousse, TUN; 2 Nutrition, Hospital University of Farhat Hached Sousse Tunisia, Sousse, TUN; 3 Infectious Control and Prevention, Hospital University of Farhat Hached Sousse Tunisia, Sousse, TUN

**Keywords:** medical disorders in pregnancy, glycated hemoglobin (hba1c), insulin, diabetes, diabetes gestational

## Abstract

The prevalence of gestational diabetes mellitus (GDM) has been steadily increasing over the past years. It is a major risk factor for glucose intolerance and type 2 DM (T2DM). The American Diabetes Association recommends that women whose pregnancy was complicated by GDM be screened for persistent glucose abnormalities at six to 12 weeks postpartum with either a fasting plasma glucose test alone or with a fasting 75-g, two-hour oral glucose tolerance test. This study aimed to identify the main predictive factors of glucose tolerance disorders in early postpartum women with a recent history of GDM. In this retrospective descriptive study, we identified 400 women who met the eligibility criteria for the study. The mean age was 34.54 ± 5.51 years. A total of 70% had a family history of DM, 16% had a personal history of GDM, and 23% had fetal macrosomia in previous pregnancies. The overall incidence of postpartum carbohydrate tolerance disorders was 36.4%, including 12% prediabetes and 24.4% DM. The prevalence of prediabetes and T2DM after delivery was higher with older maternal age, multigravidity, a higher BMI, a history of GDM, and fetal macrosomia in previous pregnancies. Furthermore, the persistence of this impaired glucose tolerance in postpartum was associated with a higher term of diagnosis, a higher glycated hemoglobin (HbA1c) percentage (the discriminant cutoff value with the best sensitivity/specificity ratio was 5.25%), the use of insulin therapy, cesarean section delivery, and fetal macrosomia. After adjusting for confounders, only prior GDM, a higher HbA1c level, macrosomia, and gestational term were found to significantly affect postpartum glucose tolerance. Although postpartum screening for T2DM is recommended for all women with GDM, a significant number of patients fail it. A better knowledge of predictive factors for this outcome is therefore needed for a more effective and targeted medical intervention.

## Introduction

Gestational diabetes mellitus (GDM) is one of the most common complications in pregnancy [1]. Its prevalence can reach 20% with the International Association of the Diabetes and Pregnancy Study Groups (IADPSG) diagnosis criteria [2]. This prevalence is steadily increasing worldwide, along with obesity and type 2 DM (T2DM). This can be related to changing dietary habits, higher maternal age during pregnancy, and increased BMI [3,4].

Although glucose tolerance usually comes back to normal in most women with GDM, the risk of persisting diabetes in the close postpartum period or ulterior diabetes development remains significant. Women with GDM were found to be seven times more likely to develop subsequent diabetes [5]. In fact, the occurrence of GDM predicts the subsequent development of T2DM with a risk of up to 50%, whereas the risk of developing carbohydrate tolerance disorders may be as high as 75% in some series [6].

According to the American Diabetes Association (ADA) guidelines, all women with GDM history should be screened for T2DM between six and 12 weeks postpartum using the 75-g oral glucose tolerance test (OGTT) [7]. However, it is unfortunate that this screening is often overlooked [8]. Epidemiological analysis found that only about 60% of women with a history of GDM received postpartum glucose testing within six months after delivery [6]. While guidelines recommend postpartum screening for T2DM in women with a history of GDM, several barriers exist that contribute to the low rates of screening, including lack of awareness, access barriers, communication gaps, and healthcare system challenges.

The identification of predictive factors for post-GDM carbohydrate tolerance disorders is therefore increasingly recommended to better target the surveillance of these at-risk women. This identification allows us to intensify the monitoring of their glucose tolerance after delivery and to initiate, if needed, preventive measures. One of the most promising stratification tools is glycated hemoglobin (HbA1c). Although it is not yet recommended for the diagnosis of GDM, it seems to be a potentially simple and effective prognostic predictor [9,10].

In the current study, we aim to determine the prevalence of carbohydrate tolerance disorders’ persistence in the immediate postpartum period and to identify their main predictive factors.

## Materials and methods

We conducted a monocentric retrospective descriptive study. We used the database records of the endocrinology department of Sousse to identify pregnant women who were treated for GDM from January 2017 to May 2020.

GDM was defined according to the diagnostic criteria of the IADPSG and the ADA [2]. This includes the presence of at least one blood glucose level during the 75-g OGTT conducted between 24 and 32 weeks of gestation, equal to or exceeding the specified threshold values: ≥92 mg/dL for fasting plasma glucose (FPG) level, ≥180 mg/dL for one-hour plasma glucose level, and ≥153 mg/dL for two-hour plasma glucose level.

In postpartum, one to three months after delivery, diabetes was diagnosed by an FPG ≥126 mg/dL or a plasma glucose level ≥200 mg/dL two hours after a 75-g oral glucose load. Prediabetes was defined as fasting blood glucose (FBG) between 100 and 125 mg/dL or a two-hour post-glucose loading level between 140 mg/dL and 199 mg/dL, according to the ADA [11,12].

We excluded from the study women with type 1 or 2 diabetes as defined by the ADA [11] or having a FBG level ≥126 mg/dL during the first pregnancy trimester. We also excluded those who did not consult within six to 12 weeks after delivery for clinical and biochemical assessment by 75-g OGTT.

Information related to the sociodemographic and clinical characteristics of the patients, the risk factors for DM, their gynecologic and medical history, and the outcomes of the pregnancy was collected. Macrosomia was defined as a birth weight of 4,000 g or more. We used the first HbA1c assay performed during pregnancy, regardless of the term at which the sample was taken. All HbA1c measurements were conducted within the biochemistry laboratory at Farhat Hached University Hospital, employing a high-performance liquid chromatography methodology. All women diagnosed with GDM underwent nutrition counseling and received personalized diets and/or insulin treatments. The ethical board committee of the University Hospital of Farhat Hached issued approval on 10/22.

Statistical analysis was performed with IBM SPSS Statistics for Windows, Version 23.0 (Released 2015; IBM Corp., Armonk, NY, USA). Clinical and biochemical characteristics of women who progressed to postpartum glucose intolerance versus women with normal glucose tolerance were compared using univariate logistic regression analysis. To define thresholds, we established a receiver operating characteristic (ROC) curve, and we chose the value with the best sensitivity-specificity combination.

In the multivariate backward regression model performed to define independent risk factors for carbohydrate tolerance disorders in postpartum, providing ORs with 95% confidence bounds, we included variables with significance levels lower than 0.2 in univariate analysis, as well as variables of known clinical interest (reported in the literature). In all statistical tests, the significance level was set at 0.05.

## Results

We identified 578 women who consulted for GDM from January 2017 to May 2020. Only 400 patients met the eligibility criteria for the study. The mean age of our patients was 34.54 ± 5.51 years, and 50% (n = 200) of the patients were older than 35 years. Among all the participants, 70% (n = 280) had a family history of DM, 16% (n = 67) had a personal history of GDM, and 23% (n = 93) had previously delivered a macrosomic newborn. The demographic and clinical characteristics associated with the glucose tolerance outcome in postpartum are displayed in Table 1 and Table 2.

**Table 1:** Demographic characteristics associated with postpartum glucose test results p < 0.05 is considered significant. DM, diabetes mellitus; GDM, gestational diabetes mellitus

Characteristics	Normal glucose tolerance	Abnormal glucose tolerance	p-value
Family history of DM (N, %)			0.425
Yes	164 (41%)	100 (25%)	
No	90 (22.5%)	46 (11.5%)	
History of GDM (N, %)			<10^-4^
Yes	28 (11.02%)	39 (26.71%)	
No	226 (88.98%)	107 (73.29%)	
History of macrosomia (N, %)			0.003
Yes	46 (18.11%)	42 (28.76%)	
No	208 (81.89%)	104 (71.23%)	
Mean age (years), mean ± SD	34.06 ± 5.49	35.38 ± 5.48	0.021
Mean gravidity, mean ± SD	2.76 ± 1.773	3.13 ± 1.82	0.049
BMI before pregnancy	26.67 ± 5.39	29.98 ± 6.49	10^-4^

**Table 2:** Clinical gestational characteristics associated with postpartum glucose test results p < 0.05 is considered significant. GDM, gestational diabetes mellitus

Clinical characteristics	Normal glucose tolerance	Abnormal glucose tolerance	p-value
Gestational age at diagnosis (weeks, mean ± SD)	23.13 ± 7.32	26.32 ± 10.39	10^-4^
Arterial hypertension (N, %)			1
Yes	5 (01.97%)	3 (02.06%)	
No	249 (98.03%)	143 (97.94%)	
Obesity (N, %)			0.001
Yes	70 (27.56%)	63 (43.15%)	
No	184 (72.44%)	83 (56.85%)	
Mean weight gain (kg)	9.32 ± 6.31	9.21 ± 6.80	0.9
Treatment of GDM (N, %)			0.001
Nutrition	77 (30.31%)	23 (15.75%)	
Insulin	177 (69.69%)	123 (84.25%)	
Delivery mode (N, %)			0.004
Vaginal	147 (57.87%)	89 (65.44%)	
Cesarean section	107 (42.13%)	47 (34.56%)	
Macrosomia (N, %)			10^-4^

Before their pregnancy, 28.9% (n = 112) of women were overweight, and 35.6% (n = 138) had obesity. The weight gain during pregnancy was above the recommended level (13) for 25.8% (n = 104) of patients.

The mean gestational age at diagnosis of GDM was 24.8 ± 6.7 weeks. During the OGTT, FPG ≥92 mg/dL, one-hour plasma glucose level ≥180 mg/dL, and two-hour plasma glucose concentration ≥153 mg/dL were detected in 63.7% (n = 209), 84.6% (n = 275), and 76.2% (n = 247) of patients, respectively.

The treatment of GDM required insulin use in a total of 280 women (70%). The cesarean section rate was 36% (n = 140), while macrosomia complicated 49.3% (n = 74) of the deliveries. The overall incidence of postpartum carbohydrate tolerance disorders was 36.4% (n = 146), including 12% (n = 48) prediabetes and 24.4% (n = 98) diabetes.

As shown in Table 1 and Table 2, the prevalence of prediabetes and T2DM after delivery increased with older maternal age, multigravidity, higher BMI, history of GDM in previous pregnancies, and history of delivering a macrosomic newborn. Furthermore, the persistence of this impaired glucose tolerance in postpartum was associated with a higher diagnosis term, the use of insulin for the treatment of GDM, the delivery by cesarean section, or the delivery of a macrosomic newborn.

As indicated in Table 3, neither FBG nor the levels of plasma glucose at one hour and two hours showed an association with the glycemic prognosis following delivery. However, a higher HbA1c percentage was significantly correlated with prediabetes and early postpartum T2DM.

**Table 3:** Glycemic laboratory characteristics associated with carbohydrate tolerance disorders in postpartum p < 0.05 is considered significant. FBG, fasting blood glucose; HbA1c, glycated hemoglobin; OGTT, oral glucose tolerance test

Characteristics	Normal glucose tolerance	Abnormal glucose tolerance	p-value
FBG (mmol/l), mean ± SD	5.61 ± 0.82	5.67 ± 0.96	0.51
One-hour glucose OGTT (mmol/l), mean ± SD	11.37 ± 7.81	11 ± 1.56	0.62
Two-hour glucose OGTT (mmol/l), mean ± SD	9.48 ± 1.41	9.15 ± 2.14	0.096
HbA1c (%), mean ± SD	5.39 ± 0.41	5.60 ± 0.50	10^-4^
HbA1c cutoff (N, %)			p = 0.005
HbA1c <5.25% (N, %)	73.3% (96)	26.7% (35)	
HbA1c >5.25%	58.7% (134)	41.3% (93)	

**Figure 1:**
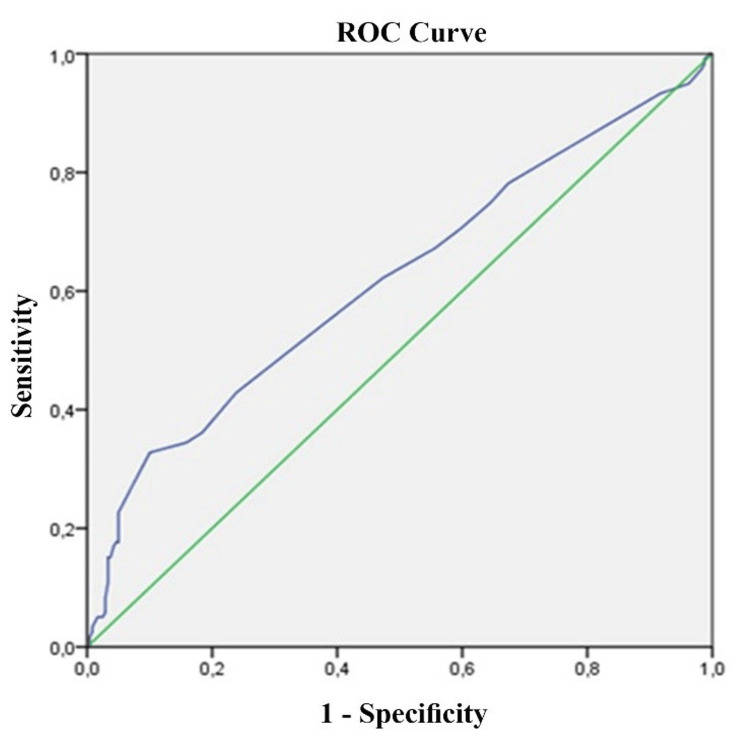
ROC curve of the specificity sensitivity for the determination of the HbA1c threshold HbA1c, glycated hemoglobin; ROC, receiver operating characteristic

In light of these findings, a ROC curve was generated to illustrate the possibility of predicting, based on the HbA1c level, the persistence of glucose intolerance after pregnancy (Figure 1). The ability of the ROC curve to predict diabetes was fair (area under the curve = 0.61, p < 10^-4^). The discriminant cutoff value with the best sensitivity/specificity combination was 5.25%.

Indeed, among the participants, 41.3% (n = 93) of women exhibiting an HbA1c >5.25% had persistent glucose intolerance in postpartum versus 26.7% (n = 35) of patients with HbA1c <5.25% (p = 0.005).

In the multiple logistic regression analysis, after adjusting for confounders, only prior GDM, higher HbA1c, and macrosomia were found to significantly affect the prevalence of postpartum glucose tolerance abnormalities (Table 4).

**Table 4:** Independent risk factors for carbohydrate tolerance disorders in early postpartum using multivariate logistic regression analysis p < 0.05 is considered significant. GDM, gestational diabetes mellitus; HbA1c, glycated hemoglobin

Risk factor	OR	95% CI	p-value
History of GDM	2.59	1.34-4.99	0.005
Macrosomia	3.21	1.76-5.86	10^-4^
HbA1c	1.98	1.37-2.85	10^-4^

## Discussion

GDM is a glucose tolerance disturbance of variable level of severity that develops during pregnancy. Its prevalence has been steadily increasing recently. In the postpartum period, it has been well documented that the risk of developing glucose intolerance, or T2DM, is significant [13,14]. Hence, it is important to identify the different clinical and biological profiles predisposed to this risk. In this sense, we conducted a retrospective descriptive study using the database records of the endocrinology department of Sousse from 2017 to 2020. A total of 400 women with GDM were included. The mean gestational age at the GDM diagnosis (by 75-g OGTT) was 24.8 ± 6.7 weeks. We identified early postpartum carbohydrate tolerance disorders in 36.4% of patients: 12% prediabetes and 24.4% T2DM. Then, we studied the related potential factors for these disorders.

In the review of the literature by Kim et al. published in 2002 [14], the prevalence of persistent diabetes after GDM ranged from 2.6% to 70%. In a more recent review by Pastore et al., the prevalence of T2DM and prediabetes varied from 1.1% to 34.6% and from 12.2% to 50%, respectively [6]. To explain this wide variation in prevalence, several reasons have been put forward, such as the preexisting higher risk of DM correlated with favorable genetics of certain ethnic groups [15], the heterogeneity of study design (retrospective and prospective), the different criteria used for the diagnosis of GDM and impaired glucose tolerance in postpartum among authors, the duration of follow-up, and, in some studies, the presence of a selection bias (no separation of preexisting and undiagnosed DM). In the reviewed researches, the magnitude of the association between GDM and the subsequent impaired glucose homeostasis, in addition to the unsatisfying rate of women participating in the recommended postpartum screening [8], suggests that more effective and targeted interventions are needed.

In the present study, the univariate analysis showed that the antepartum characteristics associated with prediabetes and DM in postpartum were age, history of GDM or macrosomia, pre-pregnancy BMI, gestational age at diagnosis of GDM, insulin therapy in pregnancy, HbA1c level, delivery by cesarean section, and macrosomia.

The independent predictive factors for the persistence of glucose tolerance disorders in women with a previous diagnosis of GDM identified by the multivariate regression were a history of GDM in previous pregnancies, macrosomia, and a higher HbA1c level. Multiple potential risk factors for prediabetes or diabetes in the early postpartum period have been described in other studies.

In 2021, a systematic review and meta-analysis including approximately 2.8 million women identified older age and family history of diabetes as two of the most common risk factors for T2DM after GDM [16]. Another meta-analysis exploring 39 relevant studies (95,750 women), including 10 studies (26%) that evaluated the risk of developing T2DM in the first year after childbirth, found that obesity, overweight, and older age were significant risk factors for progression to diabetes after GDM. Unlike the present study, a family history of diabetes was also associated with abnormal glucose tolerance (RR = 1.70 (95% CI: 1.47, 1.97); I2 = 13%) [17]. A cross-sectional study of South African women did not find a significant link between progression to T2DM and a family history of diabetes (p = 0.913) [18]. Overall, the data is not yet conclusive on whether a family history of T2DM is an independent risk factor.

In this same analysis, besides a high HbA1c percentage, increased levels of fasting, one-hour, and two-hour blood sugar tests after OGTT were associated with the risk of future glucose tolerance disorders [17]. Claesson et al. showed that the third trimester of HbA1c in the prediabetes range was associated with persistent DM [9]. In a retrospective study evaluating the HbA1c performed between 26 and 30 weeks of pregnancy and using follow-up data after delivery for 54 out of 321 women, an optimal cutoff value for HbA1c (5.5%) was defined by a ROC curve analysis as a predictor of ulterior glucose tolerance disorder. However, the limited number of women included in this analysis makes the results less reliable. In another retrospective study of 305 women, Bartakova et al. isolated an optimal cutoff value for HbA1c in mid-pregnancy of 5.4% [19]. As for first-trimester dosage, a 2022 study of 4,068 pregnant women reported a threshold of 5.4% and 99 mg/dL for HbA1c and FBG, respectively. Women with HbA1c above 5.4% were found to have a very high probability of ulterior T2DM or abnormal glucose hemostasis. Therefore, the author suggests that women with HbA1c >5.4% in the first trimester should be recalled if they miss their postpartum OGTT [20]. In our study, the cutoff value, also defined by a ROC curve, was 5.25%. HbA1c is not usually the preferred tool for glucose monitoring during pregnancy since it is not considered sufficiently efficient in capturing rapid glucose fluctuation due to metabolic and hormonal changes and increased red cell turnover during this period [21]. However, its measurement is rapid and convenient, and it is increasingly studied as an epidemiological stratification tool among women with GDM. The HbA1c in the first trimester should reflect the glycemic profile of the patient before conception. It seems plausible that even within the normal range, HbA1c is linked to the GDM outcomes postpartum.

Nevertheless, in this study, we do not always dispose of the HbA1c before the initiation of the nutritional plan or even the pharmacological treatment of GDM. Therefore, the HbA1c of many of our patients may be influenced by the treatment. In addition, while HbA1c concentration is influenced primarily by hemoglobin and plasma glucose concentration, our HbA1c levels are not interpreted in relation to hemoglobin levels or hematology abnormalities. Iron deficiency and iron deficiency anemia are very frequent during pregnancy [22]. Epidemiological and clinical studies have shown that iron deficiency anemia can induce an increase in HbA1c, irrespective of plasma glucose levels [23,24].

Unlike the results of this study, the earlier term of pregnancy at the diagnosis of GDM before increasing insulin resistance between 16 and 26 weeks [25,26] is a commonly described risk factor for progression to postpartum prediabetes, or T2DM. This association may be attributed to the possibility of preexisting undiagnosed or subclinical glucose intolerance. Meanwhile, we attribute our finding to the delay of intervention after a potential diagnostic delay.

Insulin remains the preferred pharmacologic treatment when glycemic goals are not met with nutritional modifications alone [27]. Therefore, this need for treatment with insulin can be considered an indicator of the severity of hyperglycemia. The necessity to use insulin after the GDM diagnosis is also a frequently established predictor of glucose tolerance disturbance after delivery [17,18].

In the abovementioned meta-analysis [17], the risk of developing DM was not associated with birth weight nor fetal macrosomia (RR = 1.19 (95% CI: 0.86, 1.58)) and (RR = 0.91 (95% CI: 0.44, 1.86)), respectively. In our study, this same risk was promoted by cesarean sections and macrosomia. The decision for a cesarean section is often made because of the high rate of associated complications, such as macrosomia, high blood pressure, and very high blood glucose levels [28]. The discrepancies between findings in different studies regarding the association between birth weight, fetal macrosomia, and the risk of developing DM could be attributed to several factors, including methodological differences, population characteristics, and variations in the healthcare systems or practices across different settings. Differences in study designs, such as retrospective vs. prospective studies, may lead to variations in findings. Retrospective studies rely on medical records and may be subject to biases, while prospective studies follow participants over time, allowing for better control of confounding factors. Also, differences in the prevalence of comorbidities or complications related to pregnancy, such as gestational hypertension or preexisting diabetes, could affect the risk of developing DM postpartum. On the other hand, variations in clinical guidelines or practices related to the management of pregnancy complications, such as cesarean sections for macrosomia or other indications, could influence the observed associations.

Limitation

The main limitation of our work is its retrospective nature, as we analyzed the available HbA1c levels done at random terms during pregnancy, potentially influenced by the treatment and the presence of associated anemia, as well as the lack of data regarding lifestyle habits and physical activity.

## Conclusions

Women with GDM in whom glucose tolerance disorders persisted in postpartum had different anthropometric, clinical, and biological patterns from those who had normal glucose homeostasis after delivery. An older age, history of GDM or macrosomia, high pre-pregnancy BMI, gestational age at the diagnosis of GDM, insulin therapy during pregnancy, high HbA1c (>5.25%), delivery by cesarean section, and macrosomia were linked to postpartum glucose tolerance disorders. A better knowledge of these predictive factors is needed to ensure a more targeted and early medical intervention.
